# Genomics of pain in osteoarthritis

**DOI:** 10.1016/j.joca.2013.06.010

**Published:** 2013-09

**Authors:** M. Thakur, J.M. Dawes, S.B. McMahon

**Affiliations:** Neurorestoration Group, Wolfson CARD, School of Biomedical Sciences, Kings College London Guy's Campus, London SE1 1UL, UK

**Keywords:** Osteoarthritis, Pain, Genomics, GWAS, Symptomatic, Genetics, Nociception

## Abstract

Osteoarthritis (OA) accounts for the majority of the disease burden for musculoskeletal disorders and is one of the leading causes of disability worldwide. This disability is the result not of the cartilage loss that defines OA radiographically, but of the chronic pain whose presence defines symptomatic OA. It is becoming clear that many genes, each with a small effect size, contribute to the risk of developing OA. However, the genetics of OA pain are only just starting to be explored. This review will describe the first genes to have been identified in genomic studies of OA pain, as well as the possible dual roles of genes previously identified in genomic studies of OA in the context of pain. Difficulties associated with attempting to characterise the genetics of OA pain will be discussed and promising future avenues of research into genetic and epigenetic factors affecting OA pain described.

## Introduction

Recent years have seen substantial advances in the understanding of the genetic architecture of osteoarthritis (OA). Unbiased genetic screens of large OA cohorts such as those included in the arcOGEN and TREAT-OA consortia have had sufficient power to reveal a wide range of relatively common susceptibility loci^1^. These studies have defined OA according to a composite definition, with cases either having structural damage to the joint, evaluated using radiographic criteria such as the Kellgren Lawrence score, or having previously undergone joint replacement.

However, the presence of radiographic evidence of OA does not always correlate with the main clinical presentation of OA, which is joint pain. Patients consistently describe the most distressing aspect of living with OA as the fatigue, disability and reduced quality of life produced by chronic joint pain^2^. A disease-modifying drug that restored cartilage without any reduction in pain would have little clinical utility^3^. Currently available analgesics have limited efficacy, high numbers needed to treat and high incidences of adverse events when taken chronically to combat joint pain^4^. Therefore, a greater focus on symptoms as well as aetiology of cartilage loss is critical if OA research is to produce clinically relevant therapies. Some recent genomic studies have acknowledged this by including a large proportion of patients who had previously undergone joint replacement^1^. Joint pain is likely to be the main factor prompting the decision to replace joints in these patients. But despite this fact, there have been very few studies which set out to specifically examine the genomic factors underpinning OA pain, in contrast to the relatively well characterised genomics of broadly-defined OA risk and cartilage loss.

This review will describe the genes identified in current OA genomic studies which focussed on joint pain, as well as the possible dual roles of genes identified in broader genomic studies of the disease. The difficulties and limitations of genetic and epigenetic studies will also be discussed.

## Which genes are currently known to associate with OA pain?

The gold standard for genetic association studies are unbiased Genome-Wide Association Studies (GWAS). These are well suited to the identification of common gene variants with small or large effects, but require large cohorts of patients to provide sufficient statistical power, and hence are costly to perform. An example is the £2.2 million arcOGEN study, which in its first phase compared 7410 patients with severe OA with 11,009 unrelated controls in a UK cohort, and went on to replicate the most promising signals from this cohort in a wider European cohort of 7473 cases and 42,923 controls^1^.

Although 80% of patients included had undergone joint replacement, the arcOGEN study did not set out to identify genetic influences on pain in OA *per se*, but used a focus on severe OA as a means of increasing the power of the analysis by ensuring greater homogeneity of their OA cohort. As the decision to perform joint replacement in these patients would have been informed principally by the degree of pain and disability the patients were experiencing, the presence of joint replacement can arguably function as a valid proxy for explicit measures of joint pain.

As part of its analysis, the study did look for loci that had stronger association in a joint replacement only subset of patients (i.e., those who had experienced pain associated with OA) vs the broader composite-defined cohort (which included the patients who only had radiographic changes). Four such loci were found, corresponding to chromosome regions close to the genes *GLT8D1*, *ASTN2*, *CHST11* and *TP63*, none of which are presently represented in the pain literature. Investigations of the role of these genes in preclinical models of OA pain will reveal if they can be manipulated to affect pain outcomes.

A study guided by a novel imputation strategy in the arcOGEN cohort genotyped an single nucleotide polymorphism (SNP) in *MCF2L* as a risk locus for OA^5^. *MCF2L* codes for the Rho-specific Guanine nucleotide exchange factor DBS. DBS is phosphorylated by TrkC, a receptor of the Nerve Growth Factor (NGF)-family member neurotrophin NT-3, and enhances Schwann cell migration when activated^6^. Growth factors are potent regulators of pain-sensing fibre function^7^. Antibody therapies neutralising NGF are the single most promising putative analgesics currently being assessed for OA pain^8^. *MCF2L* regulation of Schwann cell–neuronal interaction could conceivably play a role in the dysregulated articular innervation observed in osteoarthritic cartilage^9^. However, NT-3 is expressed at very low levels in synovial fluid of individuals with RA and OA compared to expression of the other neurotrophic factors NGF and brain derived neurotrophic factor (BDNF)^10^. Also, the SNP was equally frequently found in the radiographically- and symptomatically-defined cohorts used in the study. This makes it less likely that this SNP has a specific role in OA pain over cartilage loss.

To date, the only adequately powered GWAS to be carried out in the pain field was for chronic widespread pain^11^. This may reflect the difficulties in undertaking time-consuming and potentially expensive standardised quantitative phenotyping of this subjective phenomenon across a large cohort of individuals of diverse ethnic and cultural backgrounds. Accordingly, candidate gene association studies, which can be adequately powered with much smaller cohorts, are more common in the pain field. However, as these smaller studies are hypothesis-led, they tend to have a narrow focus on genes already known to be involved with pain sensitivity, and are thus more likely to produce false positive results.

One reason for the relatively small body of detailed data on pain genomics in OA is that the cohorts used for radiographic OA GWAS tend to be community-based, while symptomatic criteria tend to be better reported and assessed in clinical settings. Additionally, radiographic reporting criteria are more standardised than symptomatic reporting criteria, which frustrates the formation of large subgroups of patients with similar symptomatic profiles^12^. However, from the existing studies, there are five genes claimed to associate with OA pain.

The *SCN9A* gene encodes the alpha subunit of voltage gated sodium channel Na_v_1.7. This is selectively expressed by nociceptors and known to be essential for transmission of pain-related signals. Rare mutations in the channel confer a congenital insensitivity to pain. In contrast, gain of function is seen in primary erythromelalgia, fibromyalgia and idiopathic small fibre neuropathy^13^, ^14^, ^15^. These mutations have Mendellian characteristics. In addition, however, a SNP conferring an Arg-1150-Trp substitution in this channel was found to associate with higher pain reports in four clinical trial cohorts of 578 OA patients, as well as in a variety of other painful conditions and healthy controls. The SNP in question is present in 10% of people and was thought to account for around 0.8 points on the 20 point Western Ontario and McMaster Universities Arthritis Index (WOMAC) pain subscale^16^. Unfortunately this SNP has failed to replicate in a larger independent cohort^17^. The authors of the latter study conclude that their result may be consistent with a weak effect of the Arg-1550-Trp substitution on overall pain sensitivity, rather than a specific effect in OA, as the SNP was also present more often in individuals with poorly localised multiple regional pains.

TRPV1 is a ligand-gated ion channel enriched on thermosensitive peripheral nerve fibres. TRPV1 is expressed on articular chondrocytes^18^, as well as intraarticular nerve fibre terminals^19^, ^20^. Ile-585-val TRPV1 variants are reported to confer reduced sensitivity to cold pain^21^, ^22^. The Ile–Ile variant is associated with lower risk of symptomatic vs asymptomatic knee OA (odds ratio (OR) = 0.74) in a cohort of 3270 symptomatic vs 1098 asymptomatic cases^23^. This association was specific to symptomatic OA, and was not seen in patients with radiographic changes but no pain. There are many other SNPs in *TRPV1* which are already known to affect pain reporting^24^. It will be interesting to assess whether these other variations also have any association with symptomatic OA over asymptomatic OA.

Paired amino acid converting enzyme 4 (PACE4, coded by the *Pcsk6* gene) is an enzyme in cartilage that activates the cartilage degrading aggrecanases ADAMTS-4 and -5^25^. In a candidate gene study in 674 patients with radiographic knee OA with pain vs 2068 radiographic knee OA/no-pain patients, a SNP in *Pcsk6* was strongly associated with protection against pain when radiographic OA was present, with an OR of 1.33. While there was no further investigation to determine whether this specific SNP conferred a loss or gain of function, the group also performed a number of pain tests in *Pcsk6* null transgenic mice. These mice had, on the whole, a normal pain phenotype, with the exception of a mild reduction in mechanical sensitivity as well as substance P- and acetic acid-evoked pain behaviours^26^. Unfortunately, the phenotype of these mice in experimental OA models is not yet known.

P2X7 is a purinergic channel expressed on cells of myeloid lineage such as macrophages. A SNP in this gene determines the ability of the channel to form a pore that allows high molecular weight material to pass through the membrane. P2X7 is highly polymorphic in humans^27^. Individuals with radiographic OA (numbering 743 cases) possessing the hypofunctional variant of the channel were significantly less likely to have clinically relevant OA pain when compared with 586 unaffected controls (defined quite liberally at a WOMAC score >3 in this study – 6 is more often used as the cutoff). This study also showed that SNPs in P2X7 associated with pain intensity in a post-masectomy pain cohort. P2X7-selective antagonists are currently being evaluated as analgesics in both inflammatory and neuropathic pain states^28^, though there are currently no studies of the role of this gene in experimental OA.

Finally, the common Val-158-Met polymorphism reduces the activity of the catecholamine degrading enzyme catechol-O-methyltransferase (COMT). This SNP was shown to increase the risk of hip pain amongst 171 female, but not 288 male individuals with confirmed radiographic OA, with an OR of 4.9^29^. COMT polymorphism is also known to associate with maladaptive coping and pain catastrophising in fibromyalgia and shoulder pain^30^, ^31^, as well as influencing acute experimental pain reports in healthy controls^32^.

When assessing these studies, it is important to note the relatively small cohorts used. With one of the genes linked to OA pain already shown not to replicate in a larger cohort, it remains to be seen whether the contribution of the other genes will be confirmed or refuted by further studies. The genes linked to pain in OA are summarised in Table I.

**Table I:** Genes identified by genomic studies of symptomatic OA

Gene	Protein	Effect in OA	Study size	Reference
*Glt8d1*	Glycosyltransferase 8 domain-containing 1	More often found in cases defined purely by presence of joint replacement vs cases defined using composite measure of radiographic change and/or replacement	7410 hip and/or knee OA cases vs 11,009 controls. Approx. 80% of cases had undergone joint replacement.	^1^
*Astn2*	Astrotactin 1			
*Chst11*	Chondroitin sulfotransferase 11			
*Tp63*	Tumour protein P63			

*Mcsf2L*	Rho-specific Guanine nucleotide exchange factor DBS, phosphorylated by TrkC	SNP enriched in cases vs controls – but common in both radiographic and symptomatically-defined cohorts	19,041 cases vs 24,504 controls in meta-analysis. Approx. 80% of cases had undergone joint replacement.	^5^

*Scn9A*	Voltage gated Sodium Channel 1.7 (Na_v_1.7) expressed in peripheral nerve	Arg-1150-Trp substitution associated with higher pain reports; failed to replicate.	578 patients with symptomatic OA in original study (radiographic status omitted), 1325 ROA + Pain or TKA vs 529 ROA only in replication (KL variable)	16, 17

*Trpv1*	Transient receptor potential cation channel, subfamily V, member 1 (TRPV1), peripheral nerve nociceptor channel	Il-585-Val SNP associates with a 25% lower risk of symptomatic OA	3270 ROA + pain vs 1098 ROA only cases (KL ≥ 3 or TKA)	^23^

*Pcsk6*	PACE4, activates aggrecanases	SNP enriched in asymptomatic patients with radiographic OA	2068 ROA + pain vs 674 ROA only (KL ≥ 2)	^26^

*P2x7*	P2X purinoceptor 7, ligand-gated ion channel allowing formation of large pores on dendritic cell membranes	Hypofunctional SNPs associate with less severe pain	743 Symptomatic OA vs 586 controls (radiographic status omitted)	^33^

*Comt*	COMT, catecholamine degrading enzyme	Hypofunctional Val-158-Met polymorphism confers 30% increased risk of pain in females but not males with radiographic OA	105 ROA + pain vs 171 ROA only (KL ≥ 2)	^29^

ROA = osteoarthritis defined on radiographic criteria alone.

## Genes identified in expression studies

In addition to the identification of genes which associate with OA pain in genetic variation studies, changes in gene function can also be assessed using expression profiling. One study used expression profiling of blood leukocytes to identify a subgroup of symptomatic OA patients who overexpressed IL-1B, reported higher pain scores, and were at greater risk of radiographic progression^34^. However, very few of the existing expression profiles of osteoarthritic tissue were originally designed with pain genes in mind, and many focus exclusively on chondrocytes or matrix proteins.

The exception is a study which compared gene expression in cartilage of OA patients and healthy controls using genome-wide microarray analysis^35^. Table II presents the genes with greater than 10 fold positive or negative change in expression with a known role in pain (based on available publications on Pubmed). These amount to 35% of the total genes with >10 fold change (22/63). Many of these genes encode proteins that can activate nociceptors. In preclinical pain research, intraarticular monosodium iodoacetate (MIA) has been widely used to model OA-like joint pain. This leads to histopathological changes in rodents which have some of the features seen in osteoarthritic human cartilage, and is accompanied by robust and well defined alterations in pain-related behaviours^36^. Microarray analysis of cartilage from the MIA model found that nearly 2000 genes were significantly dysregulated, with many of these genes related to degradation and inflammatory processes^37^. However when compared to human OA cartilage, less than 4% of the differentially expressed genes were common with the rat. The reason for such a large discrepancy in gene expression is unclear but may reflect the different etiologies of the experimental model and the naturally-occurring disease.

**Table II:** Genes identified through expression profiling in osteoarthritic cartilage with >10 fold change + or −, annotated with known pain role, grouped according to biological role. Genes with no pain role not shown. OA microarray data from^35^

Upregulated	Gene	Protein name	Role in pain
Cytokines	LIF	Leukemia inhibitory factor	Anti-inflammatory and analgesic^39^, but can also induce mechanical hyperalgesia^40^

Chemokines	IL8	Interleukin 8	Hyperalgesia-inducing via sympathetic nervous system^41^, ^42^. Anti-CXCL8 serum reduces inflammatory pain^41^

	CXCL2	Chemokine (C–X–C motif) ligand 2	No effect on pain sensing^43^

	CCL2	Chemokine (C–C motif) ligand 3	Pronociceptive when upregulated in spinal cord^44^, ^45^. Induce hyperalgesia when given peripherally^46^, ^47^, ^48^. KO of receptor prevents nerve-injury induced mechanical hyperalgesia^46^.

	CXCL14	Chemokine (C–X–C motif) ligand 14	Upregulated in inflammatory pain^49^

	CCL3	Chemokine (C–C motif) ligand 2	Proinflammatory, sensitises TRPV1^50^. Induces hyperalgesia when administered locally^50^, ^51^

Enzymes	MMP13	Matrix metallopeptidase 13 (collagenase 3)	Regulated by miRNA-146a in OA pain^52^

	PDE4B	Phosphodiesterase 4B	Null mice have normal pain^53^

	JMJD3	Jumonji domain containing 3	Can alter histone methylation in response to inflammation^54^

Growth Factors	IGF1	Insulin-like growth factor 1	Underexpressed in fibromyalgia^55^

Matrix Components	TNC	Tenascin C	Interacts with voltage gated sodium channel subunit beta^56^

Bone-related	POSTN	Periostin, osteoblast specific factor	Downregulated by NSAID rofecoxib^57^

Others	RGS1	Regulator of G-protein signaling 1	Tonically antagonises opioid receptors^58^

	AQP1	Aquaporin 1	Coexpressed in Nav1.8 positive C fibres, but KO has no change in pain phenotype^59^

	PENK	Proenkephalin	Released by peripheral immune cells. KO increases pain behaviours^60^

	MARCKS	Myristoylated alanine-rich protein kinase C substrate	Phosphorylation of spinal MARCKS maintains neuropathic pain^61^

	ASPN	Asporin	Associated with lumbar disc degeneration pain^62^

	WNT5A	Wingless-type MMTV integration site family 5A	Abberant activation association with acute, chronic and inflammatory pain^63^, ^64^, ^65^.

	SFRP4	Secreted frizzled-related protein 4	Differentially regulated after nerve injury in mice with disrupted nociception^66^

Down regulated	Gene		

	FKBP5	FK506 binding protein 5	Variants predict neck pain persistence after injury^67^

	TXNIP	Thioredoxin interacting protein	Regulates diabetic neuropathy^68^

In contrast, a recent study showed that gene expression in humans and rats is highly significantly correlated in a translational model of experimental inflammatory pain^38^. These studies together suggest that, while there is little similarity in the underlying mechanisms governing the tissue degeneration process in preclinical models of OA vs clinical disease states, the pathogenesis of pain may involve substantial commonalities.

## Do any of the genes identified in genomic studies of cartilage loss in OA have known roles in pain?

A frequently cited aspect of OA is the discordance said to exist between radiographic changes in the joint and symptoms of joint pain or stiffness^69^. However, a number of recent studies have presented evidence for a greater degree of concordance between structural and symptomatic events in OA than had previously been acknowledged. This may reflect the increased use of higher resolution magnetic resonance imaging of joint tissue^70^, ^71^, ^72^ as well as greater standardisation in the reporting of radiographic findings^12^. However, it remains true at the population level that there are many patients with severe radiographic changes who are asymptomatic, as well as individuals experiencing joint pain with only minimal radiographic changes. One longitudinal study found that only 56% of patients with a Kellgren Lawrence grade (KL) ≥3 were currently experiencing joint pain^73^.

These caveats may account for the fact that very few of the existing genes linked to cartilage loss in genomic studies, such as DOT1L, have any known role in the regulation of pain^74^. However, there are two radiographic OA-linked genes that do have characterised pain phenotypes or close links to pain targets.

The first is TRPV4. A loss of function mutation in *TRPV4* was shown to be associated with inherited osteoarthropathy, suggesting a role for TRPV4 in articular cartilage homeostasis^75^. TRPV4 is known to have functions in visceral pain, transduces hypotonicity-evoked pain and is a target for a number of pro-algesic inflammatory mediators including prostaglandin E2^76^, ^77^, ^78^. However, it is currently not clear whether TRPV4 is expressed on articular nociceptors.

The second gene identified from studies of OA defined radiographically is the interleukin-1 receptor. A candidate gene study and a large meta-analysis identified an association between the C-T-A haplotype of *IL1RN*, the IL-1 receptor antagonist, and reduced severity of radiographic OA^79^, ^80^. *IL1R* knockout mice have a reduced pain phenotype^81^, and cytokines play a key role in the generation and maintenance of joint pain^82^. Unfortunately, the frequency and severity of the symptomatic OA phenotype in the populations from these studies is not reported, and the meta-analysis indicates that the effect of the *IL1RN* polymorphism on OA risk is likely to be weak^79^, ^83^.

In the field of rheumatoid arthritis, there are now a number of studies suggesting that the temporal relationship between pharmacological analgesia and disease course may differ, with onset of pain relief apparently preceding changes in joint disease in some instances^84^, ^85^, ^86^. The potential dual role of the genes discussed above begs the question of whether these factors could directly alter pain transmission, aside from any known effects on articular pathology in OA.

## The limitations of genomic studies of painful joint disorders

One potential confounding factor of the studies described above is the finding that the presence of knee pain can affect X-ray imaging of the joint in extension. This effect will occur when imaging the weight-bearing anterioposterior aspect, due to the disinclination of patients experiencing greater knee pain to fully extend the affected joint^87^. Thus changes in joint space narrowing in clinical trials and epidemiological studies of radiographic OA progression may not be entirely independent of (often unexamined) variation in the symptomatic status of the population being studied^79^, ^83^.

Another potential confounding factor is ethnic variation in pain sensitivity. Ethnic variation in genes associated with cartilage loss in OA has been repeatedly encountered, frustrating efforts to replicate promising GWAS candidates^88^. It is already clear that significant differences in pain perception exist in individuals from different ethnic backgrounds^89^. Any large-scale studies of symptomatic OA will have to take this into account.

The likely factors with the strongest influence on persistent pain in OA are illustrated by the study of OA patients with poorly controlled symptomatic OA who undergo total knee arthroplasty (TKA). Patients who will go on to require TKA are distinguished by reduced joint flexion, effusion and pain; interestingly, even severe radiographic degeneration has no bearing on the need for joint replacement^90^. Joint replacement significantly reduces knee pain reports in the majority of cases. However, it is also true that up to 44% of patients experience some degree of persistent pain 3–4 years after surgery. Patients' description of their pain indicates that, in most cases, this persistent pain is not simply due to intraoperative nerve damage. The strongest risk factors for pain persisting after knee replacement were the existence of coincident pain at other sites, and comorbidity of major depression^91^. This suggests that genes found to associate with OA pain in studies which do not correct for the presence of comorbid pain and depression in their OA cohorts may in fact be genes associated with these risk factors. For example, polymorphism in *COMT* is known to have a role in mood disorders, as well as symptomatic hip OA^29^, ^92^, ^93^.

Genomic studies of painful conditions similar in pathogenesis to OA are likely to also be relevant to OA pain. The OPPERA study of temporomandibular disorder (TMD, a chronic joint pain disorder with a large affective component) is one example. Similarly to individuals with symptomatic OA, individuals with TMD in the OPPERA study are more sensitive to pain applied elsewhere on the body and experience greater temporal summation of noxious stimuli, indicating central sensitization^94^, ^95^. TMD sufferers were also more likely to have suffered previous trauma, and had more comorbid pain conditions, like OA patients with persistent pain^91^, ^96^. The two most promising SNPs identified in the analysis of the OPPERA cohort are SNPs in *COMT*, as described above, and the serotonin receptor *HTR2A*^97^. Revealingly, SNPs in *HTR2A* are also associated with the risk of chronic widespread pain^98^.

The identification of genes regulating general pain sensitivity, as opposed to specific OA pain risk, concurs with data from twin studies and studies of *COMT* polymorphism. Taken together, these diverse studies indicate strong underlying genetic factors for general pain reporting across many body sites, as opposed to factors regulating pain reporting at specific sites or in specific conditions^32^, ^99^. *COMT* polymorphism may also determine magnitude of response to both analgesics and placebos^100^, ^101^, ^102^. This implies that genomic data may be of great utility in the design of enriched-enrolment clinical trials for symptomatic OA treatments, to maximise the differentiation of drug-treated and placebo-treated groups. But genomic data may be less successful in providing a means of tailoring treatments for individuals, other than flagging patients who are likely to require greater analgesia.

## Promising future avenues of research into genetic and epigenetic factors affecting symptomatic OA

### Endophenotypes in symptomatic OA

One way of increasing the power of genetic studies hoping to identify quantitative trait loci is to focus on endophenotypes, rather than the simple case status (in this case, the latter would be the presence or absence of radiographic/symptomatic OA). Endophenotypes are measurable intermediate biomarkers that may have a more direct relationship to a gene product^103^. An example in OA is a GWAS that identified genes linked to cartilage thickness, as defined by degree of joint space narrowing^74^.

In symptomatic OA, a focus on endophenotypes could entail subdivisions such as: low, medium and high pain intensity groups; patients whose pain persists following total knee replacement and patients whose pain is resolved; patients who experience pain localized to the joint alone and those who experience referred pain; patients who experience pain with neuropathic features and those without any neuropathic component; patients whose pain is well controlled by non-steroidal anti-inflammatory drugs (NSAIDs) and those with uncontrolled pain.

A handful of studies in the pain field are already using these stratified populations on a smaller scale to probe the neurobiology of symptomatic OA in patients and in preclinical models^91^, ^104^, ^105^, ^106^. Deployed on a larger scale, such studies could use endophenotypes to deliver more powerful and clinically relevant genomic data on the extremely heterogeneous symptomatic OA population. Endophenotype analysis is already being attempted in the OPPERA TMD cohort, and has identified potentially distinctive clusters of gene regulation in patients who experience localized vs more widespread pain^92^.

### Epigenetics

Epigenetics is the study of mechanisms that regulate gene function, which can be heritable or stabilised within an individual, without altering the DNA sequence. Examples of such include histone modification and DNA methylation. So far genetic variation studies have identified a number of relatively common candidate genes with small effects on overall risk of OA, defined radiographically. However these genes cannot fully explain the strong genetic component which is evident in OA^107^. Therefore other mechanisms must be involved – such as epigenetics.

Existing epigenetic studies have focussed on articular cartilage, where DNA methylation and histone modification have been linked to the production of catabolic factors which regulate cartilage degradation^108^. Recently, epigenetic mechanisms have also been implicated in the development and maintenance of chronic pain^109^. For instance, the direct manipulation of histone modification mechanisms through the use of histone-deacetylase inhibitors can reduce pain-related behaviours in animals subjected to an inflammatory insult^110^. It is therefore conceivable that epigenetic modifications contribute to OA pain. One possible avenue might be via the regulation of the immune system which has well characterised epigenetic components^111^. Immune cells are active players in OA pathology and can produce pro-algesic factors such as cytokines and chemokines, which likely contribute to OA pain. The production of such factors is thought to be strongly influenced by epigenetic processes^112^, ^113^.

Micro (mi)RNAs are another way in which gene function can be regulated without changing the DNA sequence. RNA interference regulates gene expression through the production of small RNA sequences (miRNAs) which can bind to target mRNAs and prevent translation. Genetic deletion of Dicer, an important enzyme in miRNA production, shows that this process is important for the ability to detect inflammatory pain^114^. In OA, miRNAs play a role in disease progression, in particular by regulating chondrocyte function, and may contribute to cartilage degradation^115^. miRNA-146a has been linked to OA progression since its expression is upregulated in OA cartilage^116^. In rats this miRNA can regulate the expression of many inflammatory genes which may contribute not only to chondrocyte dysfunction but also pain^117^. In human chondrocytes miRNA-199 has been shown to directly regulate the expression of COX-2, the rate-limiting enzyme in prostaglandin synthesis^118^. Since COX-2 is the target of NSAIDs, the first line analgesic treatment used in OA patients, it seems possible that miRNA-199 might directly influence pain sensitivity in OA through the regulation of pain mediator production, in this case prostaglandin.

## Conclusion

Recent years have seen an explosion in the volume and quality of genomic studies in the field of OA. A minority have set out to identify genes that regulate joint pain in OA. In future studies of the genetic architecture of OA pain, the use of endophenotypes and a focus on epigenetic processes will help to clarify the role of genetic and epigenetic factors in the development of OA pain. The subsequent advances in understanding should contribute greatly to the design of effective treatments which could relieve the substantial burden of OA pain.

## Author contributions

All authors contributed equally to the conception of this review and gave final approval of the version submitted. MT and JD drafted the article, SM provided critical revision.

## Role of funding source

None of the authors received any funding relating to the writing of this manuscript.

## Conflicts of interest

MT, JD and SM have no conflicts of interest to declare.
